# Refractory overactive bladder: Beyond oral anticholinergic therapy

**DOI:** 10.4103/0970-1591.32069

**Published:** 2007

**Authors:** Ronald W. Glinski, Steven Siegel

**Affiliations:** Center for Continence Care and Female Urology, Metro Urology Specialists, 2550 University Avenue West, Suite 240N, St. Paul, MN 55114

**Keywords:** Behavioral therapy, botulinum toxin type A, electrical stimulation, overactive bladder, urinary incontinence, urologic surgical procedures

## Abstract

**Objectives:**

In this review, we discuss the treatment of refractory overactive bladder (OAB) that has not adequately responded to medication therapy and we propose an appropriate care pathway to the treatment of OAB. We also attempt to address the cost of OAB treatments.

**Materials and Methods:**

A selective expert review of the current literature on the subject of refractory OAB using MEDLINE was performed and the data is summarized. We also review our experience in treating refractory OAB. The role and outcomes of various treatment options for refractory OAB are discussed and combined therapy with oral anticholinergics is explored. Emerging remedies including intravesical botulinum toxin injection and pudendal neuromodulation are also reviewed, along with conventional surgical options.

**Results:**

In general behavioral therapy, pelvic floor electrical stimulation, magnetic therapy and posterior tibial nerve stimulation (PTNS), have shown symptom decreases in 50-80% of patients with OAB. Depending on the study, combination therapy with oral anticholinergics seems to improve efficacy of behavioral therapy and PTNS in approximately 10-30%. In multicenter, long-term randomized controlled trials, sacral neuromodulation has been shown to improve symptoms of OAB and OAB incontinence in up to 80% of the patients treated. Studies involving emerging therapies such as pudendal serve stimulation suggest that there may be a 15-20% increase in efficacy over sacral neuromodulation, but long-term studies are not yet available. Another emerging therapy, botulinum toxin, is also showing similar success in reducing OAB symptoms in 80-90% of patients. Surgical approaches, such as bladder augmentation, are a last resort in the treatment of OAB and are rarely used at this point unless upper tract damage is a concern and all other treatment options have been exhausted.

**Conclusion:**

The vast majority of OAB patients can be managed successfully by behavioral options with or without anticholinergic medications. When those fail, neuromodulation or intravesical botulinum toxin therapies are successful alternatives for most of the remaining group. We encourage practitioners responsible for the care of OAB patients to gain experience with these options. More research is needed to assess the cost-effectiveness of various OAB treatments

The diagnosis of overactive bladder (OAB) has moved to the forefront of urologic practice today as it has become a widely recognized problem for a large number of urologic patients. The costs to the society are significant. The total cost of (OAB) in the United States (US) has been estimated at 12.6 billion dollars per year, with 9.1 billion incurred by people in the community and 3.5 billion noted from institutional residents.[1] It has been estimated that 16-45% of adults have OAB with or without urinary incontinence.[2]

Drugs are the mainstay of treatment for OAB in the United States. Unfortunately, current medications often have limited efficacy, resulting in incomplete resolution of OAB symptoms in a large proportion of patients.[3] Side-effects are the most important issue relating to persistence and adherence to drug therapy, even among patients who may experience symptomatic benefit.[4] It is clear that the OAB symptom complex may be due to factors other than the detrusor, including an up-regulated sensory apparatus and/or abnormal behavior of the pelvic floor muscles. It follows that there are limits to the potential efficacy of currently available drugs, which are predominately antimuscarinic agents that target the detrusor. Many patients experience concurrent bowel problems and chronic pelvic pain, which also must be addressed simultaneously. Fortunately, alternative therapies have emerged for OAB refractory to oral drugs.

In this review we specifically focus on the treatment of nonneurogenic OAB. This includes frequency/urgency and urge incontinence, although many of the treatments discussed are used for neurogenic OAB if the situation dictates. We will focus on old and new treatments for OAB. We hope the reader will embrace a broader outlook on the management of OAB, beyond oral anticholinergic therapy.

## LIFESTYLE CHANGES/BLADDER TRAINING

Lifestyle changes are often critical in controlling OAB symptoms. Behavioral management involves awareness and voluntary alteration of certain behaviors that may promote voiding dysfunction. Smoking cessation,[5] weight loss,[6] limiting caffeine intake[7] or other dietary changes can decrease symptoms. Many patients with constipation can have associated voiding complaints. Increasing fiber and fluid intake can help improve urinary symptoms in this group. The simple act of recording voiding patterns on paper may be the first step in the right direction. Once a pattern is discerned, techniques such as timed voiding to preempt an urge episode or bladder drill to lengthen intervals between voiding are helpful. Fantl *et al*, in a randomized controlled trial, showed that bladder training decreased the number of incontinent episodes by 57%.[8] These are simple things that can be done that do not cost much and may help patients better control their bladder symptoms even if they are already taking antimuscarinics.

## PELVIC FLOOR MUSCLE TRAINING-BIOFEEDBACK

Kegal exercises, as a form of behavioral therapy, have been used for years to help women control urinary incontinence,[9,10] due to the fact that the pelvic floor muscles have been noted to be the voluntary “on-off switch” for the detrusor. We use pelvic floor muscle electromyographic (EMG) biofeedback to help patients gain recognition of the location and function of the pelvic floor muscles. While verbal instruction and/or demonstration during exam may be sufficient for some, use of perineal, transanal or transvaginal EMG sensing devices can better help most patients to recognize their pelvic floor muscles. In many patients, pelvic floor muscle dysfunction is not a strength issue, but instead a range of motion and coordination problem. Therefore, therapists actively teach patients to relax the pelvic floor muscles and then to isolate and contract them in a coordinated fashion. A combination of “quick flicks” and maximum contraction and hold techniques are practiced until the patient can demonstrate improved relaxation and range of motion with increased strength and efficiency. Once accomplished, these “urge suppression” techniques are actively employed to dissociate the sense of urge from the act of voiding. As with any exercise or behavioral change, it takes time for benefit to occur (typically 6-12 weeks for most patients). The therapist plays a key role in encouraging the patient to work through this period, until symptomatic improvement is apparent. The changes in muscle function require continued exercise to maintain a benefit.

**Figure 1:**
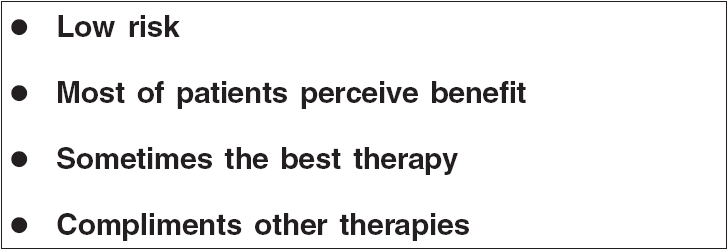
Pelvic floor muscle therapy - first line therapy for OAB

Randomized controlled trials have proven the efficacy of behavioral therapy with or without biofeedback. A 50-80% reduction in the number of stress and/or urge incontinence episodes has been demonstrated, with approximately 15-30% of women achieving near continence with behavioral therapy.[11,12] Many believe that in the era of medical cost containment and considering the billions of dollars spent on urinary incontinence, support does exist for the recommendation that pelvic floor muscle training be considered as first-line therapy in the treatment of not only urge, but stress incontinence also.[13] It is relatively cheap and offers virtually no risk to the patient [Figure 1].

Other studies explored the role of behavioral modification combined with pharmacological intervention. Mattiasson *et al* reported a 33% reduction in voiding frequency in those receiving tolterodine with bladder training compared to a 25% reduction with those on tolterodine alone.[14] Burgio *et al* demonstrated decreased rates of incontinence in 57.5% of patients receiving biofeedback alone compared to 88.5% of the patients in the group that combined biofeedback plus medication.[15] This matches our own observation that combination treatments offer greater efficacy for OAB when compared to pharmacological treatment alone. Although behavioral interventions may have a role in place of pharmacological management, they certainly can be used as an additional measure for OAB which has been insufficiently controlled by drugs alone.

Downsides of this treatment include the need for dedicated personnel to provide training and the time required not only by these personnel to train patients, but from the patient's perspective, the time required to learn the proper techniques. Patient motivation and compliance with therapy are keys to success. Because compliance can be a big issue, attrition rates have been reported to be as high as 34%.[16]

## PELVIC FLOOR ELECTRICAL STIMULATION AND MAGNETIC THERAPY

Pelvic floor electrical stimulation (PFES) and magnetic therapy (MT) both exercise the pelvic floor muscles via direct stimulation and may be advantageous for patients who cannot learn to identify the muscles by other methods. They are passive interventions that do not require active input from the patient or a therapist. However, they do not emphasize relaxation, which is an important part of pelvic floor muscle training and biofeedback. These methods can also be used as a step in teaching a patient to identify the pelvic floor, eventually enabling them to perform pelvic floor exercises independently.

Pelvic floor electrical stimulation involves placement of a small transvaginal or transanal device by the patient. Electrical stimulation for 15 min twice daily, every day or every other day is then carried out over the length of therapy. Siegel *et al*. showed 69% of 72 women with OAB using PFES were cured or improved by 50% over a follow-up period of 20 weeks. The greatest improvement came in the first six weeks of therapy, however improvement continued beyond this time interval.[17] There are few long-term studies using these devices. It is logical to assume that unless there is continued use, the benefits of this therapy will not continue. One small, long-term retrospective review of 27 women with urge incontinence showed that 78% of women complained of continued urge incontinence 10 years after maximal electrical stimulation of the pelvic floor, despite reasonable results in the short term when the original study was started.[18] It should be noted that one randomized controlled trial by Smith showed no difference between electrical stimulation and anticholinergics.[19] It appears that more studies are needed to conclusively show a long-term benefit of this therapy.

Unlike PFES, MT has the advantage of avoiding an intracavitary probe, which may be uncomfortable for the patient. Patients simply sit on a MT chair twice a week for 20min for at least eight weeks. But *et al*. showed 78.3% of the participants using MT experienced symptom improvement after two months of therapy with a mean improvement rate of 41.9%.[20] Kirschner-Hermanns *et al*. showed a 67% reduction in urge incontinence using the therapy in patients who failed anticholinergic medications.[21] Other studies have shown no benefit to MT with any incontinence syndromes including fecal incontinence.[22] In general, there are few studies available on this modality, a paucity of long-term outcomes and there are few standardized treatment protocols.

Other concerns with both of these therapies include that they require a great deal of time to administer several times per week. Like biofeedback and pelvic floor muscle training, dedicated personal may be required to administer the treatment. There is the cost of the device and finally reimbursement for these treatments, especially in the US, can be an issue. Currently, it appears that there is much better evidence to support the role of biofeedback before embarking on PFES or MT.

## SACRAL NEUROMODULATION

**Figure 2:**
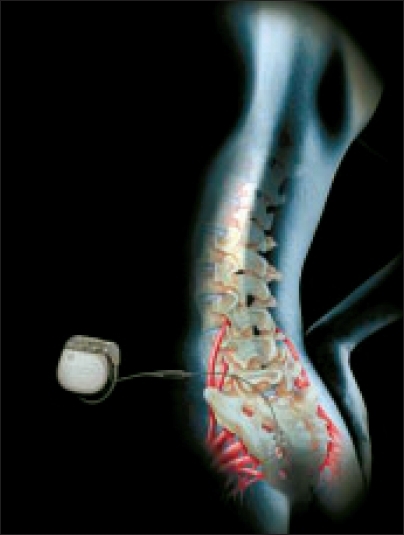
The Interstim^®^ device (Medtronic, Minneapolis, MN, USA)

In our practice, patients with bothersome OAB symptoms who have not responded adequately to conservative measures such as biofeedback and anticholinergic medications are candidates for a trial of sacral nerve stimulation (SNS). While the mechanism of action is unknown, the therapy is thought to regulate reflexes between the bladder, sphincter and pelvic floor by modulating afferent innervation.[23] This is distinctly different from drugs, which work on the motor function of the detrusor. Marketed worldwide as Interstim^®^ (Medtronic, Minneapolis, MN), SNS is a minimally invasive procedure approved for use by the FDA for urge incontinence, frequency-urgency syndrome and idiopathic urinary retention. It is performed on an outpatient basis in a two-stage format. The first stage involves placing a temporary or potentially permanent lead into the S3 foramen. During the trial, an external device is used to stimulate the lead and diaries are used to document whether there is an objective change in relevant voiding parameters. If there is a greater than 50% decrease in the patient's urinary symptoms compared to the prestimulation baseline values, a chronic lead and or implantable neurostimulator (INS) is placed [Figure 2]. If the trial is unsuccessful, the temporary lead is removed.

In a groundbreaking, multicenter, randomized controlled trial, SNS was shown to cure intractable symptoms of urge incontinence in 47% of patients and improve symptoms by at least 50% in an additional 29%, leading to a combined clinical success of 76% at six months follow-up.[24] In a second arm of the study, 56% of patients with intractable urinary frequency without incontinence were observed to return to normal urinary frequency (four to seven voids per day) or they had at least a 50% improvement in urinary frequency.[25] In a third arm, 69% of patients with idiopathic, non obstructive urinary retention were able to eliminate the use of catheters. Another 14% had a greater than 50% reduction in catheter volume per catheterization for a combined success rate among these subgroups of 83%.[26] There were no significant changes among the control groups for all three arms. Also, when the stimulation was turned off after six months of therapy, symptoms returned towards baseline parameters indicating that stimulation was necessary to alleviate symptoms. Hence, this is not a cure, but a modulator for these voiding problems.[24–26] Finally, for refractory urge incontinence, sustained benefits were noted for an average of 30.8 months (range one to five years) indicating that long-term benefit is possible from this treatment.[27]

In an 11-year retrospective review of 104 patients from our own center, Sutherland *et al* also demonstrated significant benefits of SNS for patients with intractable symptoms. For urgency/frequency and urge incontinence, mean voiding frequency dropped from 11.6 voids/24h to 8.5 voids/24h. Mean voids per night dropped from 2.2 to 1.5. Mean leaks dropped from 4.5 to 1.0 per 24h. Finally, pad use dropped from a mean of 2.3 to 0.3 pads/ 24h. All of the mean improvements in these parameters were statistically significant (*P*<0.05). Mean follow-up was 22 months (range 3-162 months). Sustained subjective improvement was >50% in 69% of the patients. Quality of life survey revealed that 60.5% were satisfied with their treatment and 16.1% were dissatisfied. Only 13% abandoned therapy during the 11-year treatment history, which is remarkable considering the dropout rate for other treatments including anticholinergic therapy.[28]

A systematic review of the current literature corroborates these findings. Brazelli *et al* reviewed four randomized controlled trials and showed that 80% of the patients in those studies achieved continence or a minimum of 50% improvement in their main incontinence symptoms. The authors also summarized 30 different case series and found 67% of the patients were similarly improved. A total of 1,827 patients with intractable urge incontinence were implanted with SNS devices in this review.[29]

Downsides to sacral neuromodulation include the cost of the device and the surgical procedure itself. Unfortunately, to our knowledge, no studies address the issue of cost for this particular procedure. However, considering that many, if not most patients with severe refractory OAB, have had these problems for several years, we believe that these costs can be made up over time. The potential improvements in quality of life that these devices can offer for many of these patients cannot be understated. Other concerns include the need for a two-staged procedure. Also, depending on the device implanted, battery life is limited to 7-10 years at best, which means that in many individuals, further surgery may be required to replace the INS. Surgical revision rates have been noted to be 33%, although generally these revisions involve relatively minor procedures.[30]

### PTNS

PTNS is a form of “percutaneous” neuromodulation. It has the advantage of being less invasive than sacral neuromodulation and offers a low risk, is easily performed and potentially less expensive treatment for refractory OAB. The posterior tibial nerve has sensory and motor components. These nerve fibers originate from L4-S3 and contain somatic and autonomic nerve supply to the pelvic floor, bladder and urinary sphincter. The tibial nerve can be accessed at the level of the medial malleolus and weekly nerve stimulations are performed for an initial period of 12 weeks. If there is benefit, a tapered treatment regimen may be implemented.[31]

In a prospective, nonrandomized, multicenter trial, 53 women with refractory OAB were treated with PTNS. Overall, 71% of the patients who completed the study were considered treatment successes. There was a 35% improvement in urge incontinence, 30% improvement in pain and a 20% improvement in incontinence-related quality of life.[32] Another study confirmed an apparent benefit of PTNS, but showed a diminished rate of success if instability symptoms were severe.[33] A recent study of PTNS in combination with low-dose oxybutynin for refractory OAB had an 83.2% response rate compared to 61.6% in the PTNS only group.[34] Although the differences did not reach statistical significance, this small study suggests a possible role of combination therapy to improve the efficacy of PTNS alone.

Based on this small group of studies, PTNS appears to be less effective than SNS, but may be attractive as a less invasive option among patients who have less intense symptoms. Unfortunately, randomized controlled trials are lacking. Posterior tibial nerve stimulation requires multiple treatments to achieve and maintain a benefit[35] and patient compliance may become an issue over time. Also, as was noted with some of the other conservative therapies already discussed, it can be time-consuming to provide weekly treatments for a large group of patients within a practice. Finally, since PTNS, like SNS, is another form of neuromodulation by direct stimulation of certain nerves, the question arises as to why it is still effective since it is only administered on an intermittent basis. As stated in the prior section, when SNS is turned off, symptoms generally return to baseline levels. Does this occur, at least partially, with PTNS? It is unclear at this point. An implantable form of PTNS which is in development may overcome some of these potential drawbacks.[31]

### Pudendal nerve stimulation

The success of SNS for control of refractory voiding dysfunction has lead to the development of new forms of neuromodulation, involving alternative sites of stimulation. The pudendal nerve has fibers originating in S2, S3 and S4. In theory, this may represent an advantage over S3 “only” sacral neuromodulation. One technique involves implantation of an Interstim^®^ lead next to the pudendal nerve through a perineal or posterior approach as a staged procedure. A recent single-center study by Peters *et al* has described the technique of pudendal nerve stimulation (PNS) and has compared it to sacral neuromodulation. Pudendal nerve stimulation was considered to be a superior lead in 79.2% of patients who were evaluated and treated for urge incontinence, urgency frequency and idiopathic urinary retention, compared to 20.8% that felt that the sacral lead was superior. Reductions in symptoms were noted in 63% of PNS patients versus 46% in SNS patients.[36] Spinelli *et al* also demonstrated success with PNS in patients with neurogenic bladder as a basis for their urge incontinence symptoms, even among those who may have had a partial or poor response with SNS.[37]

**Figure 3:**
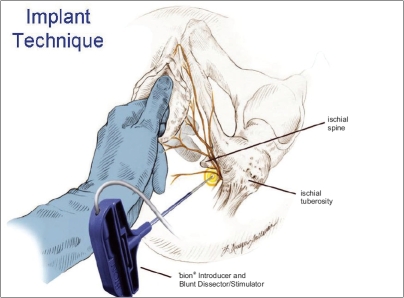
*bion*^®^ Impant Technique. This technique is considered experimental (Advanced Bionics Corp, Valencia, CA, USA)

A novel new device known as *bion*^®^ (Advanced Bionics Corp, Valencia, CA, USA) is an implantable, rechargeable system intended for chronic pudendal nerve stimulation currently under investigation for treatment of OAB.[38] The permanent device is normally placed in Alcock's canal adjacent to the pudendal nerve as an outpatient percutaneous procedure [Figure 3]. The *bion*^®^ device thus far has been used for OAB in patients where virtually all other forms of treatment were tried, including sacral neuromodulation. A recent pilot study by Groen *et al* has shown its potential usefulness in this very difficult group.[39] Further study is required to show its effectiveness.

### Botulinum toxin

Botulinum toxin (BTX) has been used for some time in the treatment of various neuromuscular disorders. Recently, it has been used urologically for OAB due to neurological and nonneurological disorders. Seven different types of BTX are known. These include A, B, C1, D, E, F and G.[40] Botulinum toxin works by inhibiting exocytic neurotransmitter vesicle formation in peripheral motor neurons. In so doing, acetylcholine release at the neuromuscular junction is prevented, thus causing paralysis of the affected muscle group.[41]

The procedure is straightforward. Using a rigid or flexible cystoscope, direct injection of botulinum toxin type A (BTX-A) into the detrusor muscle or submucosa in the bladder at various locations has been shown to directly inhibit the bladder's ability to contract autonomously in OAB patients. Our technique involves mixing 200-300 units of BTX-A with 30cc of injectable saline. Each injection site receives 1cc of BTX solution. Usually, 30 evenly distributed injection sites are chosen. Some centers inject the trigone where nerve density is greatest, whereas other centers have avoided the trigone due to concerns of inducing reflux by paralysis of the distal ureter. The technique has been modified to a 100-unit injection in the elderly or those with poor bladder contractility.[42]

Several studies have demonstrated the effectiveness of intravesical BTX as a treatment for OAB. Rapp *et al* treated 35 patients who failed anticholinergic medication for at least one month. A third had complete resolution of their OAB symptoms, 26% were slightly improved and 40% remained the same.[43] In a more recent prospective report of 100 patients with refractory OAB treated with BTX, Schmid *et al* showed an 88% improvement in bladder function, quality of life and urodynamic findings. Interestingly, only 100 units of BTX-A were used in this study. Urgency disappeared in 82% and incontinence resolved in 86% of the patients studied. Mean frequency decreased from 14 voids per day to seven. Nocturia decreased from four voids per night to 1.5. Mean bladder capacity increased from 246cc to 381cc. A striking finding was the measured change in compliance, which increased from 24 to 41 ml/cm H2O. Although this study did not look at patients with neurogenic bladder, this finding implies that treatment with BTX may be an option for patients who would otherwise need bladder augmentation to lower their bladder pressures. Side-effects and complications were low with only four patients having transient urinary retention. Only eight patients had a poor clinical benefit, but preoperatively they had extremely noncompliant bladders. Mean efficacy was noted to last for six months (± 2).[44]

Since the effects of intravesical BTX generally last for approximately six to nine months, patients require repeat injections to maintain a benefit. Urinary retention and the need to perform clean intermittent catheterization is a known side-effect of BTX bladder injections in some individuals. It is mainly because of this issue and the need for multiple procedures, sometimes twice per year, that we consider BTX injections usually after a trial of sacral neuromodulation. Its use may be contraindicated in patients with myasthenia gravis, Eaton-Lambert syndrome, amyotrophic lateral sclerosis, breastfeeding, pregnancy and in patients on aminoglycosides which could potentially act at the neuromuscular junction. Some formulations of BTX include the blood product albumin, which may be of importance to certain religious groups.[42] Resistance to BTX-A has been reported after multiple injections and a few studies have suggested that botulinum toxin type B (BTX-B) may have a role in these individuals, although duration of action appears much shorter, which could lead to increased cost with multiple injections. In a recent randomized, double-blind, placebo-controlled crossover study by Ghei *et al*, patients had significant improvements with OAB symptoms, OAB incontinence and quality of life after BTX-B injections.[45] Unfortunately, to our knowledge, no placebo-controlled studies in humans have been done for BTX-A for nonneurogenic OAB to date. Also, no standard conventions exist as to the dose or concentration of BTX to use, the number of sites to inject or what technique should be used. This is an evolving treatment that, although very promising, requires more study.

Currently in the US, it is important to discuss the fact that BTX use for OAB is an “off label” treatment for OAB and is considered investigational. Because the medication is not approved for the treatment of OAB at this point, there are issues with insurance coverage for patients and many may have a substantial “out of pocket” expense to receive this treatment. To our knowledge, only one study addressed the issue of cost with BTX-A in the treatment of OAB in the United Kingdom (UK). The conclusion of the study was that BTX-A was “highly likely to be cost-effective” in the treatment of OAB secondary to neurological and nonneurological causes when compared to standard OAB care in the UK.[46] Thus far, intravesical BTX injection for refractory OAB appears to be a safe and effective treatment without significant adverse effects.

### Surgery

Surgery remains the last resort in the treatment algorithm for OAB and in practice is performed with increasing rarity due to the success of other options previously discussed. In these cases, all other treatment options have been exhausted. Although urinary diversion can be used, most would agree that bladder augmentation is the first choice when patients get to this point. Multiple techniques for intestinal bladder augmentation have been described. With augmentation, large or small bowel is used to increase functional bladder capacity and compliance. Bladder augmentation is usually reserved for those individuals with severe detrusor instability and those at risk for upper tract deterioration secondary to a “high pressure” bladder in neurogenic and nonneurogenic disorders. These major surgical interventions include significant potential for immediate and long-term morbidity. Bowel obstruction and bladder leak are among the potential serious immediate complications that can occur. There is an increased risk of recurrent UTIs/bladder stones, delayed rupture[47] and tumor formation arising from the intestinal segment.[48] Following an augmentation, many patients will need to catheterize several times a day due to urinary retention. Most will also need to irrigate mucus that will form in the bladder regularly. Lifelong cystoscopic surveillance is also usually recommended. Of course, because of all these issues, comorbidities associated with the procedure incur cost for the patient and the medical system. Unfortunately, to our knowledge, little is written on the costs associated with these procedures. However, as stated earlier, many of these patients have no other choice to control a severely unstable bladder and in the properly chosen patient, even these major bladder reconstructive efforts can drastically improve quality of life. Many patients go on to live relatively normal lives that they would not be able to lead if nothing was done for their condition.

One procedure that has been recommended more recently is the detrusor myomectomy or autoaugmentation procedure.[49,50] This was considered an alternative to the use of bowel due to the lack of mucous production with this procedure. Also, this can be done in an extraperitoneal fashion and is relatively easy to perform. It is essentially a way to create a bladder diverticulum by removing the detrusor muscle from the dome of the bladder. This increases compliance and decreases functional uninhibited bladder contractions the same way other augmentation procedures work. One downside of the procedure is that it may not create enough bladder capacity in some individuals.[51] Also, the risk of bladder leak or rupture remains.

Denervation procedures, such as the Ingelman-Sundberg technique, have also been described to treat OAB.[52] This involves disrupting the inferior hypogastric innervation to the bladder with the intention of decreasing sensory input while maintaining motor tone. Individuals are prescreened by injecting 0.25% bupivicaine transvaginally under the trigone. If the patient has relief in the first 24h after injection, the formal procedure is then offered.[53] The dissection involves transecting the vaginal epithelium and perivesical tissue down to the plane of the bladder serosa under the trigone and is carried out laterally and posterior to disrupt the terminal branches of the pelvic nerves. Overall response rates of 68% with complete responses in 54% have been reported in rare studies of small groups of patients using this technique.[54]

Cystolysis[55] and bladder transection procedures[56] and sacral rhizotomy[57] have also been described, but are used infrequently due to lack of long-term efficacy data and associated morbidities. Also, sacral rhizotomy is typically performed by a neurosurgeon in the setting of spinal cord injury.

## CONCLUSION-OUR APPROACH TO REFRACTORY OAB

**Figure 4:**
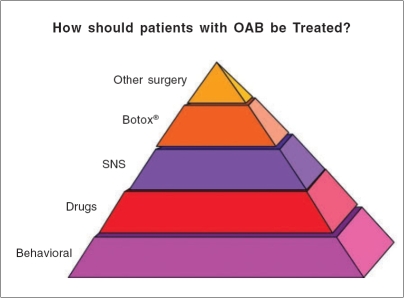
Our stepwise approach to OAB

Patients with OAB often have a significant decrease in quality of life. The cost of these treatments to society is substantial and more study in the form of randomized controlled trails is required to examine the cost-benefit ratio and effectiveness of various treatments. It is not unusual for drugs to be poorly tolerated or to show insufficient degrees of effectiveness. We believe in a stepwise approach for these patients, starting with the least invasive options first [Figure 4]. In patients who are willing and cognizant, behavioral measures including pelvic floor muscle EMG biofeedback, urge suppression techniques and bowel management with increased water and fiber intake are our preferred initial interventions. Anticholinergic drugs are offered as an alternative, but generally are used by those who are unwilling or unable to use the behavioral approach or who prefer combined therapies. In general, patients who do not respond to these conservative measures are offered other approaches. Sacral nerve stimulation is our next preferred option, due to our own large experience and the well-documented short and long-term efficacy. Posterior tibial nerve stimulation, as a minimally invasive neuromodulation approach, is also considered if this is more reasonable to the patient, although we are not convinced at this point that it is as effective in controlling significant OAB symptoms. In patients who fail to benefit from SNS, a trial of one of the forms of PNS may be offered on an experimental basis. Alternatively, intravesical BTX-A may be offered. The patient should be able to perform intermittent self catheterizations if needed. While patients may choose BTX-A over sacral neuromodulation, we feel the latter is the preferred initial option due to its proven efficacy and potential for long-term benefit. Sacral nerve stimulation also positively impacts pain and bowel symptoms, which are common among these patients. In our experience, BTX-A has no potential benefit in this regard and anticholinergic drugs typically make bowel symptoms worse. Traditional surgical solutions are very rarely used, but may include intestinal augmentation for suitable candidates. We encourage practitioners responsible for the care of OAB patients to gain experience with all these options.
